# P04 A case of zoledronate-induced ANCA associated vasculitis

**DOI:** 10.1093/rap/rkad070.025

**Published:** 2023-09-27

**Authors:** Kathryn Biddle, Laura Attipoe

**Affiliations:** Kingston Hospital NHS Foundation Trust, London, United Kingdom; Kingston Hospital NHS Foundation Trust, London, United Kingdom

## Abstract

**Introduction:**

Bisphosphonates are a well-established treatment for osteoporosis and other metabolic bone disorders. In up to 40% of recipients, infusion is associated with a transient cytokine-mediated acute-phase response. It has been hypothesised that this can rarely cause autoimmune reactions, with case reports linking bisphosphonate infusion to the development of giant cell arteritis (GCA), urticarial vasculitis and dermatomyositis. In this report, we describe a case of ANCA associated vasculitis (AAV) with 3rd nerve palsy, finger joint synovitis, fevers and cough, preceded by zoledronate infusion. To our knowledge, this is the first description of a zoledronate-induced AAV.

**Case description:**

A 75-year-old lady with a background of ulcerative colitis and gout was referred to rheumatology for the management of osteoporosis after sustaining a left neck of femur fracture. A decision was made to commence zoledronate treatment.

Within a few hours of receiving zoledronate, the patient began to feel unwell with fevers, vomiting, headache, and general malaise. Within 24 hours of the infusion she developed pain, stiffness and swelling to the small joints of her hands with fatigue, mild intermittent cough without breathlessness, and ongoing headaches. The initial impression was that she had developed a side effect to zoledronate which would be self limiting and she was treated with over the counter analgesics.

Two weeks later she remained symptomatic and bloods showed a newly raised CRP of 29 and ESR of 18. Rheumatoid factor, ANA and CCP were negative. Her joint symptoms settled with a short course of oral prednisolone, which was prescribed for a possible diagnosis of zoledronate-induced inflammatory osteoarthritis. By this point, her chest symptoms had completely resolved.

Four weeks after the zoledronate infusion, the patient developed a sudden onset 3rd nerve palsy, headache, eye pain and tingling to her face whilst in Prague. CRP was 1 and PR3 was strongly positive (131.5). MRI brain did not show any inflammation. She was treated with intravenous methylprednisolone for 6 days and her symptoms improved.

On return to the UK, she was seen by rheumatology, ophthalmology, and neurology teams. Examination showed a resolving 3rd nerve palsy, without features of retinal vasculitis or any systemic symptoms, likely due to an AAV.

She has continued on oral prednisolone and her symptoms have resolved. Her bloods show a down trending PR3 (131.5 to 23.7).

**Discussion:**

Although zoledronate is generally well-tolerated, up to 40% of patients develop transient flu-like symptoms after infusion. Commonly reported symptoms include fever, chills, muscle and joint pains and headache. These symptoms are thought to be due to a transient acute-phase response mediated by pro-inflammatory cytokines. Symptoms generally reach their peak during the first two days post-infusion, with a rapid decrease in frequency after 3 days.

In our case, the patient described many features in keeping with the well-described acute-phase response. Atypically, her symptoms were more persistent than usual with joint pain and swelling lasting for 2 weeks and an associated rise in inflammatory markers.

Following an initial improvement in symptoms with oral prednisolone, our patient developed sudden-onset 3rd nerve palsy. Investigations were in keeping with AAV and the patient responded well to corticosteroid therapy.

Although the literature is sparse, case reports and series have suggested a rare link between zoledronate and the development of autoimmune disease in patients with pre-existing risk factors . In these patients, it has been hypothesised that the acute phase response can trigger autoimmunity, however further work is needed to understand the underlying pathophysiology.

Existing literature suggests that zoledronate can trigger a range of autoimmune pathology. Narkovits et al described six patients who developed a new-onset or an exacerbation of known autoimmune disease within 3 months of zoledronate infusion. These included new-onset rheumatoid arthritis, polymyalgia rheumatica and autoimmune haemophilia with other patients reporting flares of pre-existing Crohn’s disease.

Some case reports have described the development of vasculitis secondary to zoledronate. Swarnkar et al, 2021, reported a case of zoledronate induced biopsy-confirmed urticarial vasculitis and there have been two case reports of GCA possibly triggered by zoledronate (Naderi, 2019 and Metyas et at, 2014). To our knowledge, there have been no other reports of zoledronate induced AAV.

**Key learning points:**

Zoledronate infusion is associated with a proinflammatory acute phase response in up to 40% of recipients. The most common symptoms include fevers, joint pain and swelling, gastrointestinal symptoms including abdominal pain, diarrhoea, nausea and vomiting and general features including fatigue, dizziness, headache and malaise. Very rarely, zoledronate is associated with eye symptoms including pain and scleritis.

There have been case reports linking zoledronate infusion to the development of autoimmune and autoinflammatory disease, including vasculitis. Although further work is needed to fully understand this association, clinicians should be aware of these rare reactions in order for rapid diagnosis and prompt management.

In this case, zoledronate-associated AAV responded effectively to conventional therapies with corticosteroids.

